# Intrinsic Default Mode Network Connectivity Predicts Spontaneous Verbal Descriptions of Autobiographical Memories during Social Processing

**DOI:** 10.3389/fpsyg.2012.00592

**Published:** 2013-01-07

**Authors:** Xiao-Fei Yang, Julia Bossmann, Birte Schiffhauer, Matthew Jordan, Mary Helen Immordino-Yang

**Affiliations:** ^1^Brain and Creativity Institute, University of Southern CaliforniaLos Angeles, CA, USA; ^2^Neuroscience Graduate Program, University of Southern CaliforniaLos Angeles, CA, USA; ^3^Institute of Experimental Psychology, University of DüsseldorfDüsseldorf, Germany; ^4^Faculty of Psychology, University of BielefeldBielefeld, Germany; ^5^Rossier School of Education, University of Southern CaliforniaLos Angeles, CA, USA

**Keywords:** autobiographical memory, default mode network, intrinsic connectivity, social emotion, admiration, compassion

## Abstract

Neural systems activated in a coordinated way during rest, known as the default mode network (DMN), also support autobiographical memory (AM) retrieval and social processing/mentalizing. However, little is known about how individual variability in reliance on personal memories during social processing relates to individual differences in DMN functioning during rest (intrinsic functional connectivity). Here we examined 18 participants’ spontaneous descriptions of autobiographical memories during a 2 h, private, open-ended interview in which they reacted to a series of true stories about real people’s social situations and responded to the prompt, “how does this person’s story make you feel?” We classified these descriptions as either containing factual information (“semantic” AMs) or more elaborate descriptions of emotionally meaningful events (“episodic” AMs). We also collected resting state fMRI scans from the participants and related individual differences in frequency of described AMs to participants’ intrinsic functional connectivity within regions of the DMN. We found that producing more descriptions of either memory type correlated with stronger intrinsic connectivity in the parahippocampal and middle temporal gyri. Additionally, episodic AM descriptions correlated with connectivity in the bilateral hippocampi and medial prefrontal cortex, and semantic memory descriptions correlated with connectivity in right inferior lateral parietal cortex. These findings suggest that in individuals who naturally invoke more memories during social processing, brain regions involved in memory retrieval and self/social processing are more strongly coupled to the DMN during rest.

## Introduction

It is thought that people use their own memories to evaluate and relate to other people’s social situations. Memories for personal experiences and self-relevant facts, known respectively as episodic and semantic autobiographical memories (AMs; Conway, 1992), often serve as bases from which to build mental models of others’ experiences, in order to more effectively appreciate others’ perspectives and emotional feelings (Robinson and Swanson, 1990; Ravenscroft, 1998; Frith and Frith, 2006).

Interestingly, converging evidence from neuroimaging studies reveals that many of the same neural systems that support autobiographical processing also support social processing (Svoboda et al., 2006; Schilbach et al., 2008; Mars et al., 2012; Spreng and Mar, 2012). For example, systems that support episodic autobiographical memory (Maguire, 2001) and semantic autobiographical processing, such as judgments of self-relevant traits (Kelley et al., 2002; Heatherton, 2011) and recollections of familiar, repeated experiences (Levine et al., 2004), are also involved in attributing mental states to others (Saxe and Kanwisher, 2003; Lieberman, 2007) and in feeling emotions about others’ social situations (Immordino-Yang et al., 2009; Immordino-Yang and Singh, 2011; Bruneau et al., 2012). These distributed brain systems constitute the default mode network (DMN), a functionally interconnected set of regions most consistently activated during passive rest and deactivated during tasks requiring externally focused attention (Raichle and Snyder, 2007). These regions together are thought to support the self-referential and reflective processes that are common across memory-related and social processing tasks (Buckner and Carroll, 2007; Spreng et al., 2009; Spreng and Grady, 2010; Immordino-Yang et al., 2012).

Recently, interest has grown in understanding how individual differences in the functional integrity of the DMN at rest may relate to individuals’ cognitive and emotional profiles. The distributed regions of the DMN exhibit characteristic, coherent patterns of low frequency BOLD fluctuation during awake and non-attentive task-free states (Fox et al., 2005). These intrinsic connectivity patterns are thought to reflect a fundamental property of the brain’s functional organization and have been linked with structural (anatomical) neural connectivity (Greicius et al., 2009; Honey et al., 2009). These patterns also show considerable variability across individuals and research is beginning to link this variability to task-specific neural activation/deactivation patterns (Mennes et al., 2010), and to behavioral measures of general traits, such as intelligence (Song et al., 2009), and memory ability (Wang et al., 2010). Individual differences in DMN resting state connectivity have also been associated with socio-emotional and psychological symptoms in patients with mental disorders, including depression (Greicius et al., 2007), schizophrenia (Whitfield-Gabrieli et al., 2009), autism (Assaf et al., 2010), ADHD (Uddin et al., 2008), and others. However, relations between intrinsic DMN connectivity and natural social behavior in healthy participants have not been investigated.

Given that DMN regions are known to be centrally involved in social emotional and self-relevant processing as well as in autobiographical memory, here we investigate relations between DMN intrinsic functional connectivity and individual differences in spontaneous descriptions of autobiographical memories during a natural-feeling, open-ended social emotional interview in which experiment participants describe their feelings in relation to a set of true social stories. Of note, in relation to each story presented, participants were asked to discuss their reactions in an open-ended way, but were *not* specifically asked to describe personal memories and the experimenter did not probe for memories. In this way, the autobiographical memories spontaneously described by participants during the 2 h interview are likely to reflect participants’ natural inclinations to call up memories for personal experiences in the context of social processing about unknown others’ situations.

In analyzing the memories spontaneously described by participants, we examined both episodic and semantic autobiographical memories. Both types of memories are self-relevant and acquired through life experiences. However, these memory types differ in their content and in their associated qualities of recollective experience (Brewer, 1986; Conway, 1992). Episodic autobiographical memories are memories for specific events in one’s past, and can be recalled with rich perceptual and emotional detail. Retrievals of such memories are often accompanied by a sense of reminiscence and can sometimes trigger strong emotional responses. For an example from our study, in describing how he felt after hearing a story about a young mother with cancer, one participant said, “…and I remembered how I felt when my mom got breast cancer…” and then went on to describe the details of his emotional experience.

By contrast, semantic autobiographical memories involve concepts and knowledge about one’s self that are distilled from past experiences, and recalled independent of recalling any specific past event. Such memories are generally retrieved more quickly than episodic memories (Addis et al., 2004a) and in an emotionally neutral way. For instance, in reaction to a story about a man who dislocated his elbow during a weight-lifting competition, one participant in our study said, “I have never dislocated an elbow, but I’ve lifted weights.”

Retrieval of episodic and semantic autobiographical memories is largely supported by overlapping systems that are also part of the DMN, including the hippocampi, parahippocampal gyri, medial prefrontal cortices, temporo-parietal and lateral temporal regions, and posteromedial cortices (an ensemble of cortices that includes portions of the posterior cingulate and precuneus; see Maguire, 2001; Svoboda et al., 2006; Cabeza and St. Jacques, 2007; for reviews). However, differential contributions of DMN regions to these two types of memory have also been found. Specifically, episodic autobiographical memory retrieval has been found to more heavily recruit the hippocampus, medial prefrontal, and posteromedial cortices when directly contrasted with retrieval of semantic autobiographical memories (Maguire and Mummery, 1999; Maguire and Frith, 2003; Levine et al., 2004), and the relative recruitment of these regions is thought to relate to the vividness, emotional poignancy, and self-significance of the memory (Maguire and Mummery, 1999; Piefke et al., 2003; Addis et al., 2004b; Gilboa et al., 2004; Oddo et al., 2010; see also Svoboda et al., 2006; Cabeza and St. Jacques, 2007).

In this study, we had two overarching aims: (1) to demonstrate variability across individuals in the proclivity toward describing autobiographical memories during social processing; (2) to relate this variability to individual differences in DMN regions’ intrinsic functional connectivity during rest.

## Materials and Methods

### Participants

Eighteen right-handed native English-speaking volunteers (10 females; mean age 21.2 years, SD 2.8 years; range 18–27 years), with no history of neurological or psychiatric illness, participated in the study. Participants were students or staff at a large private university on the U.S. west coast. All participants gave written consent and were compensated for taking part in the experiment. One participant identified as Latino-American, nine as Caucasian-American, six as Asian-American, and two as African-American. Data were collected as part of a larger study on neurobiological correlates of social emotions.

### Procedures

#### Social processing interview

Participants took part in a 2 h, one-on-one private video-taped interview session conducted by the same female interviewer (MHI-Y) in a quiet, dedicated room at the University of Southern California (following the method described in Immordino-Yang et al., 2009; note that the current dataset is new).

During the interview, the experimenter presented 50 narratives about true experiences of non-famous people (not actors or celebrities), some of which were meant to induce strong social emotional reactions in participants, and some of which were less emotionally evocative. The narratives unfolded like mini-documentaries, and were comprised of a scripted verbal description of 50 unique protagonists’ stories recounted (live) by the experimenter, supplemented by video and audio clips of the protagonist shown on a laptop. The narratives fell into five categories, with 10 stimuli in each: (1) Narratives involving demonstrations of marked self-sacrifice and dedication to helping others, meant to elicit admiration for virtue; (2) Narratives involving demonstrations of exceptional talents in athletics, the arts, or other domains, meant to elicit admiration for skill; (3) Narratives involving situations of bereavement, social rejection, and other forms of psychological pain, meant to elicit compassion for social pain; (4) Narratives depicting accidental bodily injuries, e.g., sports accidents, meant to elicit compassion for physical pain; and (5) Narratives involving comparable living, mentally competent people engaged in or discussing how they felt about typical activities under commonplace social circumstances. Narratives in this category were piloted to be equally interesting but relatively less emotionally evocative.

Narratives in the different categories were equivalently complex and of similar length. The corpus of narratives had been extensively piloted for emotional evocativeness, interest, cultural relevance, and other dimensions; see Immordino-Yang et al., 2009 and Immordino-Yang and Singh, 2011 for details.

The narratives were presented to each participant in one of two pseudo-random orders that counterbalanced one-back presentation history, with no more than two narratives from the same category presented in a row. After each narrative presentation, participants were asked the open-ended question, “How does this person’s story make you feel?” and were given time to answer openly. Participants were not told the emotion categories represented in the narratives, and were encouraged to talk freely and honestly about their reactions and thoughts in relation to each story. (Participants almost universally reported in a post-experiment debriefing interview that they felt comfortable and genuinely engaged during the interview, and all participants reported feeling emotional during the experiment; see Saxbe et al., 2012). Participants were not prompted for personal memories or any other information beyond the initial question.

#### Transcript coding

Videotaped interview sessions were transcribed by native American English speakers, and transcriptions were independently verified. Verified interview transcripts were edited to remove the experimenter’s speech and transcription notes, so that only participants’ responses remained for coding. Independent coders who were blind to the hypotheses judged participants’ responses to each narrative for the presence (score of 1) or absence (score of 0) of references to episodic or semantic autobiographical memories.

##### Episodic autobiographical memories

Descriptions of episodic AMs were defined as any reference to a specific event from one’s past, and the perceptual or emotional details associated with this event.

Examples:

“…this makes me especially sad just because I’m [also] gay and I’m looking for a roommate right now…”

“So I just thought of a friend of mine who committed suicide. No one had any idea because he was just incredibly successful and talented and I just wondered if he thought that… people were gonna be better off without him.”

##### Semantic autobiographical memories

Descriptions of semantic AMs were defined as any reference to autobiographical concepts or self-relevant factual knowledge that was not associated any specific event and did not contain perceptual or emotional details.

Examples:

“My family is Italian and I bake bread for a hobby myself.”

“I used to do that [skateboarding] when I was a little kid but I never had anything [any injury] like that.”

To establish coding reliability, three raters first worked together to code six transcripts from participants who were part of an earlier study, whose data were not included in this study. After this initial training period, the three raters independently coded transcripts from six participants included in this study (Fleiss’ kappa = 0.75); discrepancies were resolved by three-way discussion. The remaining 12 transcripts were independently coded by one rater; 20% of those data were randomly selected for blind verification by a second rater (Cohen’s kappa = 0.97). Because inter-rater reliability was very high for these transcripts, analyses were performed on the original rater’s codes.

Because the focus of the current study was individuals’ trait-level differences in reliance on these two types of memories, participants’ responses to narratives from the five narrative types were combined. For each participant, we calculated the number of narrative responses (out of 50) in which at least one memory was reported. Frequencies were calculated separately for episodic and semantic autobiographical memories.

#### Functional neuroimaging data acquisition and preprocessing

Approximately 50 min after the interview ended, participants underwent a 5 min resting state MRI scan, during which they were asked to “relax and rest as we take pictures of your brain.” (The data for this study were acquired as part of a larger functional study on social emotions. The resting state scan analyzed here was acquired after we acquired two 9-min functional runs in which participants viewed again the narratives they had discussed during the interview. There was a 30 min lapse between the end of the interview and the start of scanning.). Whole brain images were acquired using a Siemens 3 Tesla MAGNETON TIM Trio scanner with a 12-channel matrix head coil. Resting state scans were acquired using a T2* weighted Echo Planar (EPI) sequence (TR = 1.5 s, TE = 30 ms, flip angle = 90°) with a voxel resolution of 3 mm × 3 mm × 4.5 mm. Thirty-two continuous transverse slices were continuously acquired to cover the whole brain. Anatomical images were acquired using a magnetization prepared rapid acquisition gradient (MPRAGE) sequence (TI = 900 ms, TR = 1950 ms, TE = 2.26 ms, flip angle = 7°) with an isotropic voxel resolution of 1 mm.

Data were preprocessed using SPM8 (Wellcome Department of Cognitive Neurology, London, UK) in MATLAB 2011b (MathWorks, Inc.). Functional images were slice-timing corrected, motion corrected, and co-registered to the anatomical image. Anatomical images were segmented and normalized to MNI space (Montreal Neurological Institute) using tissue probabilistic maps (segmentation, SPM8). The same normalization transformation was applied to the functional images, which were then resampled into a resolution of 2 mm × 2 mm × 2 mm and smoothed using a 4 mm full-width, half-maximum Gaussian kernel.

#### Identifying the default mode network for each participant

The DMN was separately identified for each individual using spatial independent component analysis (ICA) carried out via the Infomax algorithm (Bell and Sejnowski, 1995) from the GIFT toolbox^1^. This software separates each participant’s fMRI data into independent (uncorrelated, non-Gaussian) spatial components and their corresponding time courses (McKeown et al., 1998). We allowed the software to estimate the optimal number of components (using minimum description length criteria; Li et al., 2007), which ranged across participants from 15 to 25.

A two-stage procedure was performed to identify the component for each participant that best corresponded to the DMN. First, we excluded components whose high frequency power (>0.1 Hz) constituted more than 50% of the total power of the component. Then, a spatial correlation was performed between each of the remaining components and a binary DMN template (mask) provided in the GIFT toolbox. (This template covers regions previously reported to be part of the DMN, e.g., Raichle et al., 2001; Buckner et al., 2008). The component that was most strongly correlated with the template was chosen as the DMN component. Visual inspection confirmed that the identified component for each participant did include the brain regions associated with the DMN. (Note that this method effectively removes from the analysis components corresponding to global signal and noise, making it unnecessary to implement band-pass filtering or to regress out global signal; see De Luca et al., 2006; Seeley et al., 2007. In addition, the connectivity of each voxel to the overall network was not affected by application of the mask or by the selection process.)

**Figure 1:**
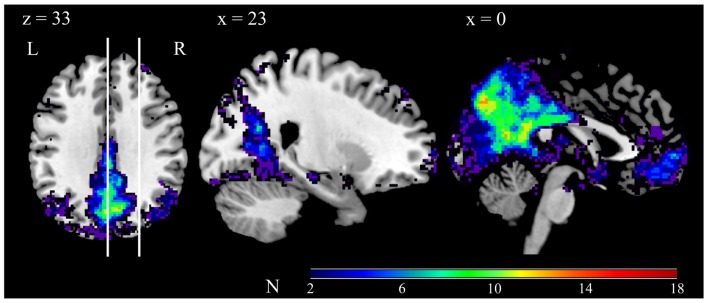
**Conjunction of the 18 participants’ DMN component maps**. Each participant’s DMN component map was thresholded at *z*-score ≥ 2, and then converted into a binary map (1 for above threshold, 0 for below threshold). Color codes indicate the degree of overlap, as per the scale depicted in the bar. The vertical lines in the left panel indicate the position of the sagittal slices. Note that the views depicted are taken from the same slice position as the views depicted in Figure 2.

We performed spatial normalization on the component map identified as corresponding to the DMN, in essence adjusting the overall strength of each participant’s DMN component in order to perform a group-level analysis. This procedure transformed each voxel’s value to a *z*-score that represents the degree to which the voxel’s time course is modulated by the time course of the participant’s overall DMN component (McKeown et al., 1998). Figure 1 depicts the conjunction of the 18 participants’ DMN component maps.

We implemented the ICA method rather than a seed-based analysis for two reasons. First, recent analyses comparing seed-based and ICA calculations of functional connectivity show that connectivity strength obtained using seed-based calculations is equivalent to the sum of within-network ICA-derived connectivity and between-network ICA-derived connectivity for a given region (Joel et al., 2011). We were interested in isolating connectivity associated only with the DMN. Second, a seed-based analysis would require that we choose *a priori* regions of interest, which would bias the results depending on the precise size and location of the ROI.

#### Correlating memory scores with DMN intrinsic connectivity

**Figure 2:**
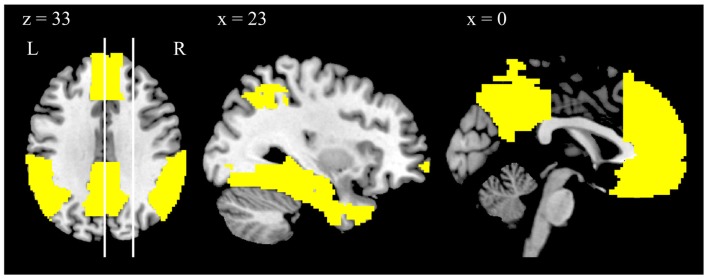
**Depiction of the anatomically defined DMN mask, displayed on a template brain**. The vertical lines in the left panel indicate the position of the sagittal slices. Note that the views depicted are taken from the same slice position as the views depicted in Figure 1.

We separately regressed on the *z*-score maps participants’ scores for frequency of episodic and of semantic AM descriptions. We then anatomically masked the whole-brain results to increase statistical power by reducing multiple comparisons. The mask was pre-defined using the Automated Anatomical Labeling Atlas (Tzourio-Mazoyer et al., 2002) to include precuneus, posterior cingulate cortices, medial prefrontal cortices, inferior parietal lobules, angular gyri, middle temporal gyri, temporal poles, hippocampi, parahippocampal gyri, and fusiform gyri; see Figure 2.

We imposed on the group-level results a statistical threshold of *p* < 0.005 and a cluster extent threshold of 23 voxels, which corresponds to *p* < 0.05 controlling for multiple comparisons. The cluster extent threshold was determined by 10,000 Monte Carlo simulation iterations conducted using the AlphaSim program in AFNI^2^. The criteria input to AlphaSim were: uncorrected *p*-value of 0.005, voxel size of 2 × 2 × 2, spatial smoothing kernel of 4 mm, and the number of voxels in the mask (48420 voxels).

#### Using bootstrapping to validate the robustness of the results

For each cluster that survived thresholding, *z*-scores from included voxels were extracted and averaged for each participant using the MarsBar toolbox in SPM (Brett et al., 2002). The averaged *z*-score and corresponding memory frequency score from each participant were paired. These pairs were randomly resampled with replacement to generate 10,000 bootstrapped samples of 18 pairs of values each, corresponding to the number of participants in the experiment. A correlation coefficient for each bootstrapped sample was calculated, and from the distribution of coefficients a 99% confidence interval was derived (Matlab version 2011b; MathWorks, Inc; see Yarkoni, 2009). For none of the identified clusters did the 99% confidence interval cross zero; hence all are reported as results.

## Results

### Behavioral results

All participants spontaneously reported semantic AMs to at least one narrative out of 50 (*M* = 6.6, Range: 2–16, SD = 3.3); 14 participants (77.8%) reported episodic AMs to at least one narrative (*M* = 2.5, Range: 0–8, SD = 2.4). Frequency of episodic and semantic AMs were uncorrelated [*r*(16) = 0.11, *p* = 0.67]. There were no effects of gender or age (lowest *p* > 0.17). Participants’ responses averaged 77 words (SD = 27; individuals’ averages ranged from 29 to 121 words).

### Correlations between AM frequencies and intrinsic DMN connectivity

**Figure 3:**
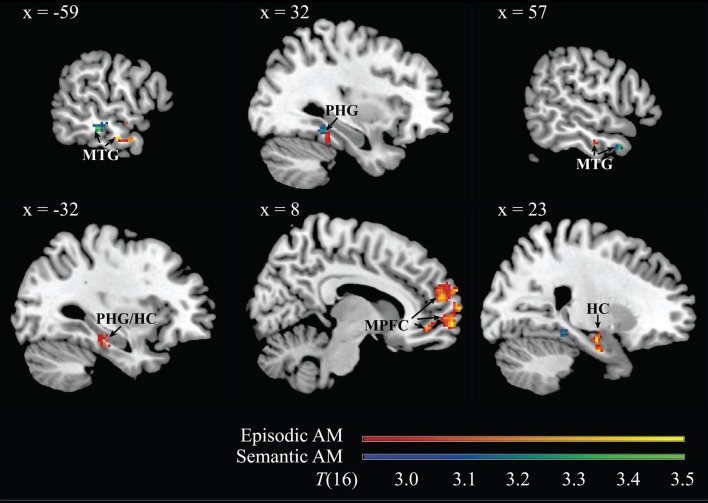
**Representative images of neural regions from within the DMN whose intrinsic functional connectivity to the overall DMN component correlated with individual differences in frequency of spontaneous verbal descriptions of episodic (red → yellow) and semantic (blue → green) autobiographical memories during the social processing interview**. Results are thresholded at *p* < 0.05, corrected for multiple comparisons. The 99% confidence interval for all depicted clusters does not cross zero. MNI coordinate of the sagittal plane is given. Note: MTG, middle temporal gyrus; PHG, parahippocampal gyrus; MPFC, medial prefrontal cortex; HC, hippocampus.

Higher frequencies of episodic and semantic AMs were linked to higher DMN intrinsic functional connectivity in middle temporal and parahippocampal gyri. Frequency of episodic AMs was additionally related to connectivity in the hippocampi bilaterally, and to connectivity in the dorsal, anterior, and ventral sectors of the medial prefrontal cortex. Frequency of semantic AMs correlated with connectivity in the right inferior parietal lobule. Neither memory type was correlated with connectivity in the posteromedial cortices (medial parietal or posterior cingulate cortices). See also Table 1 and Figure 3.

**Table 1:** **Voxel clusters whose intrinsic connectivity correlates with frequency of reported episodic (A) and semantic (B) autobiographical memories**.

Brain region	Coordinates			Cluster size	*z*-Score	99% CI of rho
	*x*	*y*	*z*	
**A. EPISODIC AM**						
dMPFC	−10	40	50	55**	4.36	[0.52, 0.97]
aMPFC	10	66	10	337**	3.76	[0.21, 0.96]
vMPFC	14	44	−8	86**	3.68	[0.26, 0.96]
MTG	−64	−6	−8	24	3.82	[0.30, 0.98]
	−60	−14	−20	28	3.20	[0.17,\; 0.94]
	64	−8	−26	58**	4.32	[0.04, 0.95]
PHG/Hippocampus	−30	−22	−20	24	3.85	[0.24, 0.97]
PHG	36	−34	−18	52**	3.68	[0.35, 0.98]
Hippocampus	20	−10	−20	56**	3.65	[0.42, 0.96]
**B. SEMANTIC AM**						
pIPL	48	−70	36	57**	3.85	[0.13, 0.99]
MTG	−64	−26	−8	69**	3.34	[0.37, 0.97]
	58	10	−26	23	3.49	[0.10, 0.97]
PHG	26	−36	−10	79**	3.46	[0.33, 0.99]

dMPFC, dorsal medial prefrontal cortex; aMPFC, anterior medial prefrontal cortex; vMPFC, ventral medial prefrontal cortex; MTG, middle temporal gyrus; PHG, parahippocampal gyrus; pIPL, posterior inferior parietal lobule. Coordinates of the peak voxel are given in MNI space. Clusters are significant at *p* < 0.05, corrected for multiple comparisons; those significant at *p* < 0.001 are marked**. Corresponding 99% confidence intervals are given (*CI* of *rho*) and do not cross zero.

## Discussion

Autobiographical memories play an important role in our social lives by allowing us to cognitively and emotionally relate to others’ situations based on simulations we build from our own memories for similar experiences and feelings. Autobiographical memories and social processing are supported by a largely shared set of neural systems (Lieberman, 2007; Buckner et al., 2008; Spreng et al., 2009), whose activity is most reliably heightened and shows functional coordination during passive rest (the DMN; Raichle and Snyder, 2007). Although there is strong interest in probing the psychological correlates of interpersonal variability in these regions’ coupling during rest, relations to individual differences in natural social behavior have not been studied. Here we provide data supporting a relationship between intrinsic connectivity in regions of the DMN known to especially support memory processing (Maguire, 2001; Svoboda et al., 2006; Cabeza and St. Jacques, 2007), self-related processing (Heatherton, 2011), and simulation of hypothetical and future scenarios (Gilbert and Wilson, 2007), and individuals’ tendencies to spontaneously describe autobiographical memories as they react to unknown others’ social situations in an open-ended interview. The results provide modest evidence that correspondences exist between natural social behavior and resting brain function.

Critical to our study design is the natural feel of the interview, and the fact that we did not probe for memories or prompt participants to describe personal experiences. We made this methodological decision to maximize the chances that our findings would reflect participants’ natural behavioral inclinations, and would relate to individual differences in social behavior outside of the lab. Indeed, over the course of the interview all participants reported feeling genuinely emotional, and outside researchers who have viewed the videotaped interviews in the context of other analyses have been struck by participants’ comfort and openness, and by their genuine reflectiveness and engagement with the narratives (S. Schnall, personal communication, June 2012), many of which recount quite extraordinary circumstances and accomplishments.

Every participant spontaneously reported at least one AM during the 2 h interview. However, there was a considerable amount of variability in the frequency with which participants described these memories, and descriptions of episodic memories were comparatively rare. Frequencies of semantic and episodic autobiographical memory descriptions were uncorrelated, and we found that these types of AM were associated with connectivity in some shared and some distinct DMN regions. Both types of AMs were associated with connectivity in middle temporal regions, which are critically involved in semantic processing, and important in the storage and retrieval of personal knowledge about the world (Martin and Chao, 2001; Binder et al., 2009). Both were also associated with connectivity in the parahippocampal gyrus. However, only episodic AMs were related to connectivity in the hippocampus, perhaps due to this structure’s role in more complex scene reconstruction (Hassabis and Maguire, 2007, 2009), and to its increased involvement in processing of memories with greater personal significance and vividness (Addis et al., 2004b; Gilboa et al., 2004; Moscovitch et al., 2005).

In addition to finding correlations between memory descriptions in the interview and connectivity in canonical memory-related regions in the brain, i.e., the hippocampus and parahippocampal gyrus, our most prominent, extensive results are in the medial prefrontal cortex. Descriptions of episodic autobiographical memories were strongly associated with connectivity in the dorsal, anterior, and ventral MPFC sectors. This region is especially involved in self-referential processing (Heatherton, 2011) and in mentalizing (Frith and Frith, 2006), and its involvement in functional studies has been shown to differentiate episodic autobiographical memories from other laboratory memory tasks without personal relevance (e.g., remembering a list of objects; Gilboa, 2004). More recently, activity in this region has been shown to be load-dependent during tasks involving working memory for social information (Meyer et al., 2012). In another experiment, MPFC activity as participants’ viewed entertaining video clips (TV pilots) predicted participants’ subsequent recall of the videos’ details when attempting to persuade another person of the videos’ entertainment value (Falk et al., in press). Analyses of the dynamic interactions between the MPFC and the medial temporal lobe system during experimentally induced episodic autobiographical recall reveal that activation in MPFC initiates and maintains activation in the medial temporal lobe (St. Jacques et al., 2011). These findings together suggest that the MPFC has an important role in strategically encoding and recalling memories with a social purpose. Our findings accord well with this interpretation, and extend previous work by demonstrating that resting connectivity in MPFC is related to the prominence of episodic memories in participants’ social processing.

Notably, we did not have significant results in the posterior cingulate or precuneus, both regions with important roles in AM retrieval (Wagner et al., 2005; Svoboda et al., 2006). It has been shown that during episodic autobiographical recall, MPFC and hippocampus were more active during the initial search and construction of the memory, whereas precuneus was more active during the elaboration phase, after the memory was successfully accessed (Cabeza and St. Jacques, 2007; Daselaar et al., 2008). It is possible that because our coding system identified the initiation of memories but did not code for the memories’ elaboration or vividness of imagery, that we did not pick up on individual variability in these dimensions that might have corresponded to medial parietal connectivity strength at rest. In a related study in progress, we find that participants who engage higher level cognitive construals of social situations, in effect more abstract, elaborated conceptualizations (Liberman and Trope, 2008), show stronger activation in posteromedial cortices during subsequent social emotion processing (Pavarini et al., under review). Future studies could investigate possible relations between individuals’ tendencies to recall autobiographical memories more vividly and elaborately, and intrinsic connectivity of posterior medial nodes of the DMN.

Our findings do seem to be specific to the DMN component of intrinsic connectivity, and are not explainable by differences in the strength of emotion participants reported experiencing in relation to our narrative stimuli. To test the specificity of the results to the component identified as the DMN, we identified the component corresponding to the salience network (anchored by orbital frontoinsular cortices and dorsal anterior cingulate, extending into ventral tegmental area; Seeley et al., 2007; Menon and Uddin, 2010) and repeated the analysis. We found no relationships between AM scores and strength of salience network connectivity in the regions in which we report DMN findings. (That analysis did reveal that a region in the superior-most portion of the precuneus showed correlation between semantic AM score and connectivity to the salience network.)

To test the relation between AM score and participants’ reported strength of experienced emotion, we utilized participants’ reports of the strength of their experienced emotion during the scanning experiment (i.e., during the functional runs that preceded the resting state scan). During the functional runs, participants viewed short versions of the video narratives they had discussed during the interview, and reported for each narrative the strength of their real-time reaction from 1 (no strong reaction) to 4 (overwhelmingly strong reaction). We correlated participants’ average reported strength of emotion with their total AM score, and found no relationship (*p* = 0.42).

Although our study, in addition to several others, have now reported correlations between individuals’ resting state connectivity and psychological traits, it is unclear at this stage how these differences should be interpreted. Our behavioral (interview) study shows a trait-level effect (tendency to spontaneously describe AMs) that is expressed in the context of a particular state (social processing). But in the neural data, trait-level and state-level effects cannot be disentangled (and may, in fact, be co-dependent). It is possible that the relations to resting DMN connectivity revealed in our study reflect differences in the resting properties of the brain that are invariant within a person. It is also possible that they may reflect either residual influences of the prior social processing task (“state” residue), or differences in the quality of thoughts individuals spontaneously engage during rest. (That is, for our study, it is possible that people who described more memories in the interview also reminisced more during the resting state scan; revealing a mind-level “trait.”). Given that individuals better at remembering past experiences also show greater intrinsic DMN connectivity (Wang et al., 2010), and given our finding that greater production of descriptions of episodic memories during social processing is related to greater connectivity at rest in neural regions supporting memory and self-relevant processing, future studies should investigate DMN intrinsic connectivity in different experimental contexts, and relations to individual differences in content of resting thought, as well as relations to social skillfulness.

Although the present analysis investigated trait-level differences among participants across emotion states, we note that the five narrative conditions did not produce equivalent descriptions of memories. Instead, although AMs were described in response to all five narrative types, disproportionately more (33%) AMs were described in response to the narratives that involved relatively commonplace, less emotionally potent social circumstances. This finding suggests that participants may have called up personal memories more often in response to stories that described situations they (arguably) would have been more likely to have personally experienced.

We also note that the quality of autobiographical memories participants recounted was aligned with an important dimension of the narrative protagonists’ situation. Our narratives had been designed to engage processing about concrete, physical circumstances separately from processing about abstract, inferred aspects of the protagonists’ psychological situation. That is, feeling compassion for another’s physical pain or admiration for another’s skill relies on straightforward perceptions of bodily actions whose emotional consequences are immediately apparent. (For example, one needs only to see the narrative protagonists’ leg break to appreciate that he is experiencing noxious physical pain). By contrast, feeling compassion for social pain or admiration for virtue requires making complex inferences about the protagonists’ hidden qualities of mind that may not be apparent from outward, observable behavior (Immordino-Yang, 2010, 2011). Interestingly, we find in the current analysis that participants recounted more semantic AMs when responding to narratives about protagonists’ physical actions or abilities (i.e., narratives meant to induce compassion for physical pain or admiration for skill; *t* = 4.6, *p* < 0.001), and trended toward producing more episodic AMs when responding to narratives about protagonists’ mental qualities, predicaments, or accomplishments (i.e., narratives meant to induce compassion for social pain or admiration for virtue; *t* = −1.72, *p* = 0.10). There were no differences in the number of AMs described in response to compassion-inducing narratives as compared to admiration-inducing narratives (approximately 33% of total memories in each case). In relation to research on experimentally induced recall of AM via interviews (e.g., Maguire and Mummery, 1999; Levine et al., 2002) or responses to word prompts (e.g., Bayley et al., 2003), this finding suggests that differences in the framing of social contexts (i.e., concrete, action-oriented versus abstract, psychologically oriented) may be an important factor shifting participants’ memories between semantic and episodic varieties.

Our study demonstrates the viability of relating natural behavior during social processing to individual differences in resting brain function, and suggests a promising future avenue for research on the neural correlates of psychological traits (see also Saxbe et al., 2012). However, our attempt to attain real-world validity also comes at a cost: participants’ verbal reports of memories may only be a subset of the memories that actually occurred to them. Reports of personal memories were relatively rare in our study. However, we believe that the number of memories participants reported would be in proportion to the total memories that occurred to them, and therefore that these numbers would reflect real individual differences in reliance on memories during social processing. As we develop a better understanding of individual differences in the contribution of memories to social processing, future research could control for the possibility that some people may be more inclined to verbally report their memories while others keep them private. Nonetheless, our findings corroborate the idea that AM is involved in social cognition during natural behavior, and extend the current literature by demonstrating that individual differences in reliance on memory processing are related to the intrinsic organization of the DMN.

## Conflict of Interest Statement

The authors declare that the research was conducted in the absence of any commercial or financial relationships that could be construed as a potential conflict of interest.

## Footnotes

^1^http://mialab.mrn.org/software/#gica

^2^http://afni.nimh.nih.gov/afni/
